# Radiomics Models Based on Magnetic Resonance Imaging for Prediction of the Response to Bortezomib-Based Therapy in Patients with Multiple Myeloma

**DOI:** 10.1155/2022/6911246

**Published:** 2022-09-05

**Authors:** Yang Li, Ping Yin, Yang Liu, Chuanxi Hao, Lei Chen, Chao Sun, Sicong Wang, Nan Hong

**Affiliations:** ^1^Department of Radiology, Peking University People's Hospital, Beijing, China; ^2^Peking University Institute of Hematology, Peking University People's Hospital, Beijing, China; ^3^Pharmaceutical Diagnostics, GE Healthcare, Shanghai, China

## Abstract

**Purpose:**

To identify significant radiomics features based on MRI and establish effective models for predicting the response to bortezomib-based regimens.

**Materials and Methods:**

In total, 95 MM patients treated with bortezomib-based therapy were enrolled, including 77 with bortezomib, cyclophosphamide, and dexamethasone (BCD) and 18 with bortezomib, lenalidomide, and dexamethasone (VRD). Based on T1-weighted imaging (T1WI) and T2-weighted imaging with fat suppression (T2WI-fs), radiomics features were extracted and then selected. The random forest (RF), *k*-nearest neighbor, support vector machine, logistic regression, decision tree, and Bayes models were built using the selected features. The predictive power of six models for response to BCD and VRD regimens were evaluated. The correlation between the selected features and progression-free survival (PFS) was also analyzed.

**Results:**

Four wavelet features were correlated with BCD treatment response. The six models all showed predictive power for BCD regimen (AUC: 0.84-0.896 in the training set, 0.801-0.885 in the validation set), and RF performed relatively better than others. Nevertheless, all the BCD-based models were incapable of predicting the VRD treatment response. The wavelet-HLH_firstorder_kurtosis was also associated with PFS (log-rank *P* = 0.019).

**Conclusion:**

The four wavelet features were valuable biomarkers for predicting the response to BCD regimen. The six models based on these features showed predictive power, and RF was the best. One wavelet feature was also a survival-related biomarker. MRI-based radiomics had the potential to guide clinicians in MM management.

## 1. Introduction

Multiple myeloma (MM) is a malignancy of plasma cells originating from the bone marrow, and most commonly present with hypercalcemia, renal failure, anemia, and bone lesions, leading to significant impairment in quality of life and placing an immense burden both on patients and society [1, 2]. In the 1980s, high-dose chemotherapy and stem-cell rescue (ASCT) was introduced as an effective treatment modality of MM, and the treatment continues to evolve rapidly with the arrival of new classes of antimyeloma drugs such as immunomodulatory drugs and proteasome inhibitors [3, 4]. Since most patients with MM ultimately relapse and become unresponsive to currently available treatment options, thus resulting in shorter survival, durable and deep remission is the key objective of MM therapy [5].

Bortezomib, a typical proteasome inhibitor, is widely used in the induction, consolidation, and maintenance therapy of MM [6, 7]. Combining bortezomib with other agents such as IMIDs, alkylating agents/doxorubicin, and dexamethasone is the backbone for doublet/triplet regimens [7]. A systematic review assessed the prognosis effects of bortezomib and demonstrated its benefit in terms of survival and response rate of MM [8]. However, part of MM patients achieved suboptimal or no response to bortezomib and may even suffer notable side effects like peripheral neuropathy and thrombocytopenia [9]. Therefore, identifying predictive biomarkers prior to MM treatment would help to avoid ineffective therapy and further optimize clinical patient management.

Some disease, host, and therapy-specific features such as performance status, tumor burden, and cytogenetic abnormalities have been reported to provide prognostic information [10, 11]. Nevertheless, all these factors and existing risk stratification systems help with prognosis for survival, and clinicians still lack reliable predictive biomarkers of treatment response [11]. In addition to clinical assessment, imaging evaluation is also an essential part of MM diagnosis and treatment, and MRI has been widely accepted as the optimum imaging modality. Since traditional imaging has difficulty detecting the treatment-mediated changes, functional imaging was recommended by many researchers [12]. However, previous studies demonstrated the ability of functional parameters for monitoring MM treatment response, their predictive capacity was limited, and response prediction remains a challenge [13, 14].

Radiomics-derived features quantify phenotypic characteristics of medical imaging, which contribute to produce accurate and robust predictions in survival, treatment response, and other clinical outcomes [15]. Many published prediction models confirmed the excellent predictive ability of CT or MRI radiomics for treatment response in cancer research, and these radiomics-based models either outperformed the existing predictive modalities or filled the gap of response prediction that has not been achieved in the clinic [16–19]. Considering previous promising results of radiomics and the advantages of MRI for bone marrow assessment in patients with MM, it is reasonable to speculate that MRI-based radiomics may have potential predictive powers for MM treatment.

In the present study, we aimed to explore the value of MRI-based radiomics for response prediction in MM patients treated with bortezomib-based therapy.

## 2. Material and Methods

### 2.1. Patients

The Institutional Ethics Committee of our hospital approved this retrospective study, and the requirement for informed consent was waived. The 357 MM patients who underwent lumbar MRI at the initial diagnosis in our hospital between January 2015 and January 2021 were preliminarily included. Inclusion criteria are as follows: (1) Patients were newly diagnosed with MM and had no previous systematic chemotherapy or radiotherapy. (2) MRI examination included sagittal T1-weighted imaging (T1WI) and T2-weighted imaging with fat suppression (T2WI-fs). (3) All patients were treated with bortezomib-based induction therapy with 3-4 cycles, and efficacy was evaluated every 2 or 3 cycles. Patients who were combined with other malignant diseases (*n* = 3), were treated with other chemotherapy regimens (*n* = 82), had previous systematic chemotherapy or radiotherapy (*n* = 56), were without complete T1WI and T2WI-fs examination (*n* = 31), had less than three cycles of induction chemotherapy (*n* = 57), and were without regular treatment response assessment at every 2 or 3 cycles (*n* = 17) were excluded. Finally, 111 patients met the criteria. Of these, 77 patients were treated with bortezomib, cyclophosphamide, and dexamethasone (BCD); 18 with bortezomib, lenalidomide, and dexamethasone (VRD); 9 with bortezomib, thalidomide, and dexamethasone (BTD); and 7 with bortezomib, doxorubicin, and dexamethasone (PAD). Considering that the number of patients treated with BTD and PAD were small, and these two regimens were less widely used in clinical practice, we included only the patients treated with BCD and VRD for further study.

Patients were followed until May 2021. PFS was defined as the time from diagnosis to the date of disease progression, death from any cause, or the latest follow-up.

### 2.2. Treatment and Response Assessment

For the BCD regimen, patients received bortezomib 1.3 mg/m^2^ on days 1, 4, 7, and 10; cyclophosphamide 300 mg/day on days 1-5; and dexamethasone 20-40 mg/day on days 1–4, 7, and 10. Each cycle of induction therapy was 21 days. For the VRD regimen, patients received lenalidomide 25 mg every other day; bortezomib 1.3 mg/m^2^ on days 1, 4, 7, and 10; and dexamethasone 20-40 mg/day on days 1–4, 7, and 10. Each cycle of induction therapy was 28 days.

In accordance with International Myeloma Working Group (IMWG) guidelines that was based on monoclonal protein level in serum and urine, the treatment responses of MM were categorized as complete remission (CR), very good partial remission (VGPR), partial remission (PR), stable disease (SD), or progressive disease (PD) [20]. Our study defined the CR and VGPR as good response and PR, SD, and PD as poor response.

### 2.3. MRI Protocol

Baseline MR images were performed on a 1.5 T scanner (Signa Excite, GE Medical Systems), 3.0 T scanner (Discovery 750, GE Medical Systems), and 3.0 T scanner (Discovery 750w, GE Medical Systems). The scan parameters were described as follows: sagittal T1WI: repetition time (TR) = 405 − 843 msec, echo time (TE) = 7.1 − 8.1 msec, slice thickness = 4 − 5 mm, matrix = 300 × 256, and field of view (FOV) = 32 × 32 cm; sagittal T2WI-FS: TR = 2500 − 3000 msec, TE = 85.3 − 125.3 msec, slice thickness = 4 − 5 mm, matrix = 300 × 256, and FOV = 32 × 32 cm.

### 2.4. Evaluation of Conventional MRI Patterns for MM

The bone marrow infiltration patterns of each patient were interpreted in consensus by two radiologists with 5 years and 13 years of experience, respectively. When there was discordance between the readers, a senior radiologist with 25 years' experience made the final decision.

The five recognized infiltration patterns are listed as follows: (1) normal appearance of bone marrow despite minor microscopic plasma cell infiltration, (2) focal involvement, (3) homogeneous diffuse infiltration, (4) combined diffuse and focal infiltration, and (5) “salt-and-pepper” pattern [12, 21]. For the quick and complete assessment of all patterns, a combination of a T1WI and T2WI with fat suppression should be employed [21].

### 2.5. Radiomics Analysis

#### 2.5.1. Image Preprocessing, ROI Segmentation, and Feature Extraction

All the eligible images were imported to the Artificial Intelligence Kit software version 3.3.0 (AK, GE Healthcare) for preprocessing, including resampling the image into 1 × 1 × 1 mm^3^, bias field correction, signal smoothing by a Gaussian filter with the standard deviation of 0.5, and intensity standardization by *z*-score normalization [22].

**Figure 1:**
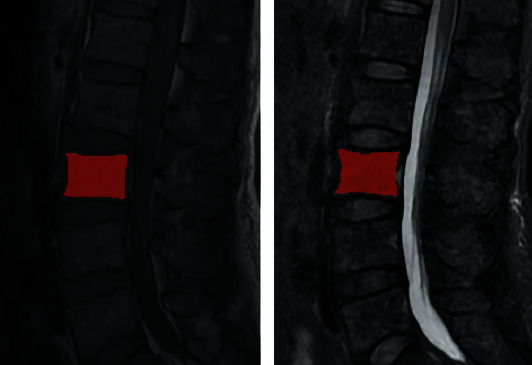
ROI segmentation.

The regions of interest (ROIs) were segmented by using the ITK-SNAP software v.3.6.0 (http://www.itksnap.org/) [23]. In our study, the entire bone marrow of the second lumbar vertebra was designated as the target region to avoid the discrepancy of the anatomical structure and ensure the stability of results [14]. The ROI was manually segmented by a radiologist with five years' experience; meanwhile, the cortical bone and degenerative changes were carefully avoided.  Figure 1 shows the ROI segmentation. Then, all the segmented ROIs were validated by a senior radiologist with 13 years of experience.

Based on AK software, there were a total of 1316 radiomics features extracted from the second lumbar vertebral body, including 18 first-order histogram features, 14 shape features, 75 texture features, and 1209 second-order features generated from the derived images via wavelet transformation, local binary pattern transformation, and Laplacian of Gaussian transformation.

#### 2.5.2. Feature Selection and Radiomics Model Construction

**Figure 2:**
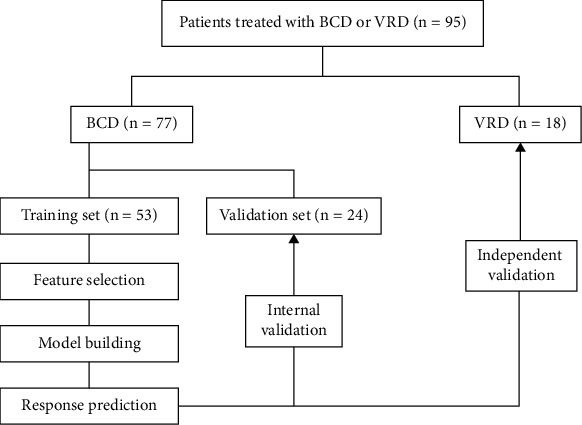
The process for MM response prediction of bortezomib, cyclophosphamide, and dexamethasone (BCD) and bortezomib, lenalidomide, and dexamethasone (VRD).

In the present study, 77 patients treated with BCD were used for the feature selection, model establishment, and internal validation, and 18 patients with VRD regimen were independently validated by the BCD-based model. The process of MM response prediction is illustrated in Figure 2.

Seventy-seven patients treated with BCD were randomly divided into a training set (*n* = 53) and a validation set (*n* = 24) at a ratio of 7 : 3. All the extracted features were firstly normalized before feature selection. The outliers were replaced with the median of the particular variance vector. Moreover, the data were standardized by *z*-score transformation. The standardized formula is as follows: (fi − *u*)/std, where fi represents a single characteristic data, *u* is the average value of the data column, and std pertains to the standard deviation of the data column. In order to simplify the model and increase the interpretability and stability of the model, we used a combination of three commonly used methods to select features, including Pearson correlation analysis (threshold, 0.7), variance threshold method (threshold, 1) and the least absolute shrinkage and selection operator (LASSO).

Based on the selected features, the logistic regression (LR), support vector machine (SVM), Bayes, *k*-nearest neighbor (KNN), decision tree (DT), and random forest (RF) models were built for MM response prediction. All the models were trained by applying the repeated fivefold cross-validation technique in the training set; then, the performance of models was evaluated in the validation set. The predictive ability was compared by using the DeLong test. Finally, the VRD regimen group was independently validated by the models above.

#### 2.5.3. The Potential Association between the Radiomics Features and PFS

For each of the selected radiomics features, the optimum cutoff value was determined by the Youden index in ROC analysis. Then, the patients were assigned into two groups by using the cutoff values. The PFS was compared between the two groups in each of the selected features.

### 2.6. Statistical Analysis

The clinical characteristics and MRI patterns of MM were evaluated by the chi-square test. The receiver operating characteristic (ROC) analysis was conducted to assess the model's predictive power, and the area under the curve (AUC), sensitivity, specificity, and accuracy were all calculated. The DeLong test was applied for model comparison. Kaplan-Meier curve analysis and log-rank tests were used to analyze the correlation between radiomics features and PFS. All statistical analyses were performed using R software (version 3.5.1) and SPSS (version 24.0). A two-sided *P* value < 0.05 was considered significant.

## 3. Results

### 3.1. Patients

Among the 95 selected patients, 36 patients were IgG type, 33 were IgA type, 4 were IgD type, 20 were light chain type, and 2 were nonsecretory type. For treatment response, 53 patients were classified as good responders and 42 as poor responders. All the clinical characters and MRI patterns had no significant differences between the good and poor response groups, as summarized in Table 1.

### 3.2. The Selected Radiomics Feature and Models' Predictive Ability

Based on the training set of the BCD regimen, four significant radiomics features were identified. Two of them were extracted from T1WI and the other two from T2WI, and all these features were wavelet transformed. The odds ratio (OR) of each feature was calculated by multivariate logistic regression. The details are presented in Table 2.

**Figure 3:**
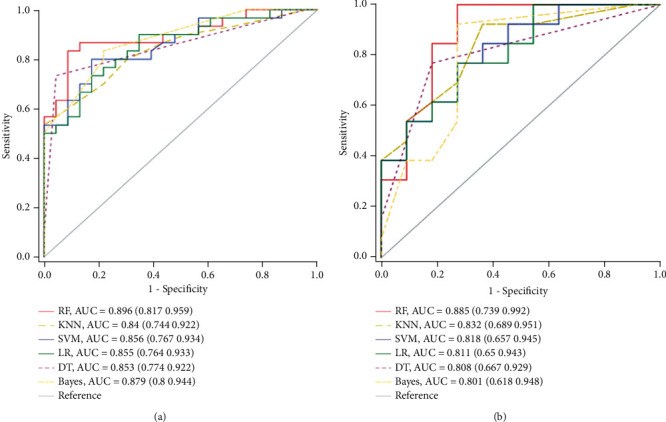
Receiver operating characteristic (ROC) curves of random forest (RF), *k*-nearest neighbor (KNN), support vector machine (SVM), the logistic regression (LR), decision tree (DT), and Bayes models for BCD response prediction. (a) The ROC curves in the training set. (b) The ROC curves in the validation set.

The LR, SVM, Bayes, KNN, DT, and RF models were constructed. The ROC curves are drawn in Figure 3, and the RF model showed the higher AUC value both in the training (0.896) and validation set (0.885) when compared with others, but the differences did not reach statistical significance (*P* > 0.05). Meanwhile, the accuracy, sensitivity, and specificity of each model were also calculated (Table 3). The confusion matrices of different models were shown in supplementary materials (available here).

### 3.3. Independent Validation of VRD Regimen

**Figure 4:**
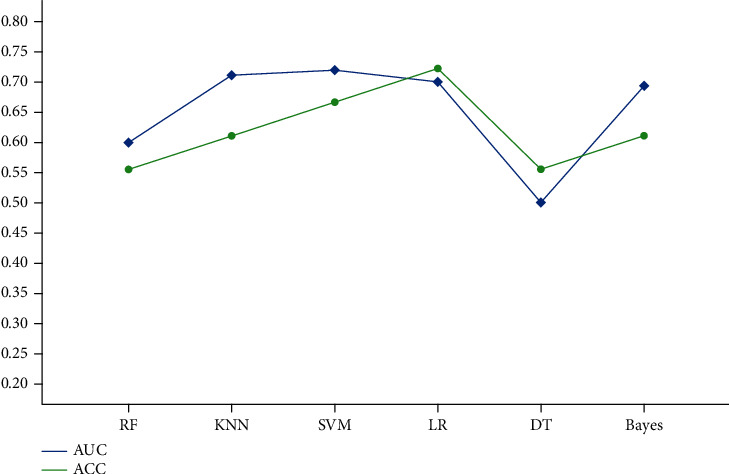
Scatterplots for the area under the curve (AUC) and accuracy (ACC) depiction of RF, KNN, SVM, LR, DT, and Bayes models to predict VRD response.

The response to the VRD regimen was independently validated by the six models constructed based on the BCD regimen. The AUC range was 0.500-0.719, and the ACC range was 0.556-0.722 (Figure 4).

### 3.4. The Relationship between the Selected Features and PFS

**Figure 5:**
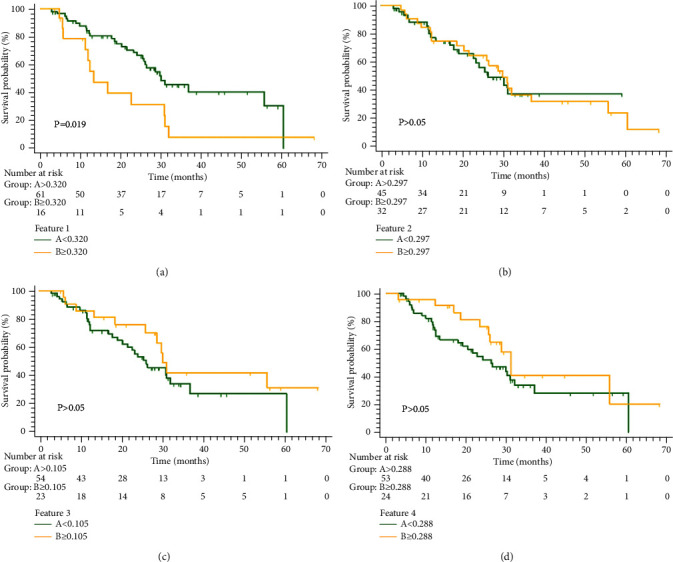
Kaplan-Meier progression-free survival (PFS) analysis of four selected wavelet features. (a) The PFS analysis of feature 1. The PFS of groups A (<0.320) and B (≥0.320) showed a significant difference. (b–d) The PFS analysis of features 2, 3, and 4. The PFS showed no significant difference between the A and B groups of each feature. Features 1, 2, 3, and 4 were wavelet-HLH_firstorder_kurtosis, wavelet-HLH_glcm_correlation, wavelet-HHL_firstorder_kurtosis, and wavelet-LLH_firstorder_mean, respectively.

For patients treated with BCD regimen, median PFS was 29.72 months. According to the Youden index, the optimum cutoff values of the four selected radiomics features (wavelet-HLH_firstorder_kurtosis, wavelet-HLH_glcm_correlation, wavelet-HHL_firstorder_kurtosis, and wavelet-LLH_firstorder_mean) were 0.320, 0.297, 0.105, and 0.288, respectively. For wavelet-HLH_firstorder_kurtosis, the PFS was significantly different between the two groups (Figure 5(a)), and the other three features indicated no difference between the two groups (Figures 5(b)–5(d)).

## 4. Discussion

In the present study, four wavelet-transformed features extracted from the MRI were confirmed as predictive factors for BCD treatment response in MM. Based on the selected features, six predictive models were built, and the RF model holds relatively more predictive power than the other models in both training and validation groups, though not statistically significant. Meanwhile, all the models lost their predictive capacity when the VRD regimen set was independently validated. Finally, the wavelet-HLH_firstorder_kurtosis was correlated with the PFS, which may be regarded as a relevant biomarker for both the BCD response and survival in patients with MM.

Although achieved PR or better represents therapeutic effectivity, our study defined CR and VGPR as good response and PR, SD, and PD as poor response for further analysis [24]. With the application of new drugs such as bortezomib and lenalidomide, MM patients usually achieved at least PR [25]. Moreover, a previous study showed that at least VGPR after induction could improve progression-free survival [26]. Achieving only PR may not satisfy the clinical expectations entirely. Some studies noted that the quality of response was not a validated effective surrogate for overall survival, but undertreatment could lead to failure of the potential cure and further progression with loss of disease control [25, 27]. Furthermore, a study demonstrated that the lenalidomide maintenance improved the prognosis, particularly among the patients who did not achieve VGPR [28]. Therefore, the achievement of VGPR has a significant implication for clinical practice.

There is an urgent need to predict the efficacy of treatment, but no promising biomarkers are universally accepted at present. Although some studies confirmed the predictive power of gene expression profiles, the results remain controversial [11, 29, 30]. The Durie-Salmon staging system, International Staging System (ISS), and Revised-ISS were commonly used for risk stratification and treatment guidance, but all these staging systems performed predictive ability for survival but were not predictive of response to therapy [10, 31]. In our study, we also found no association of staging systems between the good and poor response groups, and bone marrow plasma cell percentage ≥ 60% that reflects high tumor burden also had no ability to distinguish the quality of response. In addition, our study showed that the MRI patterns had no relationship with response to therapy. This result was reasonable, for the previous meta-analysis demonstrated that the MRI patterns were associated with survival [32], but no study indicated a correlation between MRI patterns and treatment response.

In recent years, several studies have explored the role of CT or MRI radiomics on MM treatment evaluation, the results showed good performance of radiomics, but a limitation was mentioned that patients who underwent different treatment regimens were not comparable [33, 34]. In our study, the commonly used BCD and VRD regimens were selected and analyzed separately in order to provide more reliable results. For MRI-based radiomics, Ekert et al. [33] explored the logistic model in MM treatment response assessment and reported AUC values of 0.60-0.84 based on different sequences. Our study explored and compared six predictive models both in the training and validation set; the results confirmed their predictive power, which may serve as a more comprehensive reference for future research. In addition, previous studies confirmed the efficiency of diffusion-weighted imaging (DWI) and dynamic contrasted enhanced- (DCE-) MRI [13, 14]. Therefore, the functional imaging-based radiomics could be explored in future research. However, the resolution of DWI was limited, and the DCE-MRI was rarely used due to the renal impairment in MM. The routine T1WI and T2WI were still the most frequently used for MM examination, and the radiomics based on routine sequences in our study may have the potential for better clinical practicability and higher clinical application value.

Interestingly, the four radiomics features selected from the T1WI and T2WI were all wavelet transformed high-dimensional features, which indicated that the radiomics features for MM response prediction might be more complex, and wavelet features may play a crucial role. In a previous study for rectal cancer chemotherapy response prediction, the majority of the predictive features were wavelet transformed, and authors also cited many studies with other purposes such as prediction for lymph node metastasis to stress the importance of wavelet features [16]. In a recent study for predicting the response of osteosarcoma, the wavelet transformed features also accounted for most of the proportion, and researchers speculated that the wavelet features might be more sensitive for treatment prediction [19]. To our knowledge, apart from ours, there was only one previous study that utilized the MRI radiomics for MM treatment assessment [33], but the wavelet transformed features were not included for analysis, and in-depth explorations were needed in future studies.

As shown in some studies, different machine learning models could influence the predictive performance of radiomics [35, 36]. Nevertheless, there was no consensus on which would be better. Our study constructed six predictive models, including RF, KNN, SVM, LR, DT, and Bayes, and all of them were confirmed as effective methods in many previous radiomics reports [17–19, 36]. As the results showed, all these models had the predictive ability since the AUC values in each of them were greater than 0.80 in both training and validation groups. Additionally, the RF model outperformed the other five models, although this result was not statistically significant. The radiomics models were recommended for BCD response prediction. Moreover, other effective methods such as deep learning should be explored with a large sample in the future.

Though the established models exhibited some predictive capacity for response to the BCD regimen, all these models were incapable of predicting the treatment response of the VRD regimen. To some extent, it was interpretable that some studies indicated that the effect of VRD was superior to the BCD regimen [37, 38]. Clinically, there were many patients who achieved poor response with BCD, but the replacement with VRD improved the efficacy of treatment. Therefore, our study analyzed the two regimens separately, despite both of them commonly using bortezomib-based therapy in clinical practice. In addition, the result may also be affected by the smaller number size in the VRD group. It is necessary to continue collecting data for VRD-specific predictive model building in the future. Ultimately, other regimens such as BTD and PAD were not studied due to the small sample size and the limited practical clinical application.

Aside from the prediction of response to bortezomib-based therapy, our study also analyzed the correlation between the four selected wavelet features and PFS in patients with MM and confirmed that the wavelet-HLH_firstorder_kurtosis was associated with PFS. Our research was not the first to explore the connection between treatment response-related radiomics and survival. As reported in some other cancer studies, the radiomics score calculated based on selected radiomics features for response prediction was also survival-associated [18, 39]. In contrast to these studies, we explored the relationship between each of the selected features and PFS and then identified that one feature was associated with survival, while the other three were only treatment response-related features with no survival significance. The confirmed feature that correlated with both treatment response and survival may allow clinicians to develop more individualized treatment strategies for MM patients.

There were several limitations in the present study. First, the inevitable selection bias of retrospective design. Second, the sample size was small, and multicenter studies with a large sample size were required. Third, all the ROIs were manually delineated. Further study should explore the method for automatic segmentation to avoid this laborious and time-consuming process. Lastly, the parameters of the models were optimized according to experience or experimental adjustment, which may not be the most effective.

In conclusion, our study provided six effective models based on four significant wavelet features for predicting the response to the BCD regimen, which was of great value as none of the clinical characters or conventional MRI patterns had the predictive ability. Meanwhile, one wavelet feature was also found to correlate with MM survival. MRI-based radiomics had the potential to guide clinicians in MM management.

## Figures and Tables

**Table 1:** Clinical characteristics and MRI patterns of patients.

Variable	Good responders (*n* = 53)	Poor responders (*n* = 42)	*P* value
Age ≥ 65 (years)	14	15	0.328
Sex			
Female	18	16	0.676
Male	35	26	
BMPC ≥ 60%	11	9	0.566
Treatment			
BCD	43	34	0.593
VRD	10	8	
D-S staging			
II	6	5	0.588
III	47	37	
ISS staging			
I	12	7	0.643
II	18	13	
III	23	22	
R-ISS staging			
I	10	7	0.834
II	33	25	
III	10	10	
MRI pattern			
Normal	9	7	0.191
Focal	4	6	
Diffuse	31	18	
Focal and diffuse	4	9	
Salt-and-pepper	5	2	

BMPC: bone marrow plasma cells; D-S: Durie-Salmon staging system; ISS: International Staging System; R-ISS: Revised International Staging System.

**Table 2:** Selected radiomics features and their odds ratio.

Feature	Sequences	OR (95% CI)
Wavelet-HLH_firstorder_kurtosis	T1WI	0.448 (0.229 -0.877)
Wavelet-HLH_glcm_correlation	T1WI	3.784 (1.818 7.876)
Wavelet-HHL_firstorder_kurtosis	T2WI	2.842 (1.328 6.081)
Wavelet-LLH_firstorder_mean	T2WI	4.340 (1.538 12.244)

glcm: gray level cooccurrence matrix; OR: odds ratio; CI: confidence interval.

**Table 3:** The comparison of different models.

Model	Training set				Validation set			
	AUC	ACC	SEN	SPE	AUC	ACC	SEN	SPE
RF	0.896	0.868	0.867	0.870	0.885	0.833	0.923	0.727
KNN	0.840	0.755	0.800	0.696	0.832	0.792	0.923	0.636
SVM	0.856	0.792	0.800	0.783	0.818	0.708	0.769	0.636
LR	0.855	0.755	0.700	0.826	0.811	0.708	0.692	0.727
DT	0.853	0.830	0.733	0.957	0.808	0.792	0.769	0.818
Bayes	0.879	0.792	0.800	0.783	0.801	0.833	0.923	0.727

AUC: area under the curve; ACC: accuracy; SEN: sensitivity; SPE: specificity; RF: random forest; KNN: *k*-nearest neighbor; SVM: support vector machine; LR: logistic regression; DT: decision tree.

## Data Availability

The data used to support the findings of this study are included within the article.

## Supplementary Materials

Supplementary Materials The supplementary material shows the confusion matrices for the different models.
