# Supplementary material for: Radiomics Models Based on Magnetic Resonance Imaging for Prediction of the Response to Bortezomib-Based Therapy in Patients with Multiple Myeloma
Source: Biomed Res Int. 2022 Sep 5;2022:6911246. doi: 10.1155/2022/6911246 (PMC9467708; doi:10.1155/2022/6911246)
Supplement: Supplementary Materials — The supplementary material shows the confusion matrices for the different models. [file 6911246.f1.docx]

Supplementary Materials

1. Supplementary tables.

Table S1 The confusion matrix.

|  | Predicted positive | Predicted negative |
| --- | --- | --- |
| Actrual positive | TP | FN |
| Actrual negative | FP | TN |

TP, true positive; TN, true negative; FP, false positive; FN, false negative.

Table S2 The confusion matrix of RF in training set.

|  | Predicted positive | Predicted negative |
| --- | --- | --- |
| Actrual positive | 26 | 4 |
| Actrual negative | 3 | 20 |

Table S3 The confusion matrix of RF in validation set.

|  | Predicted positive | Predicted negative |
| --- | --- | --- |
| Actrual positive | 12 | 1 |
| Actrual negative | 3 | 8 |

Table S4 The confusion matrix of KNN in training set.

|  | Predicted positive | Predicted negative |
| --- | --- | --- |
| Actrual positive | 24 | 6 |
| Actrual negative | 7 | 16 |

Table S5 The confusion matrix of KNN in validation set.

|  | Predicted positive | Predicted negative |
| --- | --- | --- |
| Actrual positive | 12 | 1 |
| Actrual negative | 4 | 7 |

Table S6 The confusion matrix of SVM in training set.

|  | Predicted positive | Predicted negative |
| --- | --- | --- |
| Actrual positive | 24 | 6 |
| Actrual negative | 5 | 18 |

Table S7 The confusion matrix of SVM in validation set.

|  | Predicted positive | Predicted negative |
| --- | --- | --- |
| Actrual positive | 10 | 3 |
| Actrual negative | 4 | 7 |

Table S8 The confusion matrix of LR in training set.

|  | Predicted positive | Predicted negative |
| --- | --- | --- |
| Actrual positive | 21 | 9 |
| Actrual negative | 4 | 19 |

Table S9 The confusion matrix of LR in validation set.

|  | Predicted positive | Predicted negative |
| --- | --- | --- |
| Actrual positive | 9 | 4 |
| Actrual negative | 3 | 8 |

Table S10 The confusion matrix of DT in training set.

|  | Predicted positive | Predicted negative |
| --- | --- | --- |
| Actrual positive | 22 | 8 |
| Actrual negative | 1 | 22 |

Table S11 The confusion matrix of DT in validation set.

|  | Predicted positive | Predicted negative |
| --- | --- | --- |
| Actrual positive | 10 | 3 |
| Actrual negative | 2 | 9 |

Table S12 The confusion matrix of Bayes in taining set.

|  | Predicted positive | Predicted negative |
| --- | --- | --- |
| Actrual positive | 24 | 6 |
| Actrual negative | 5 | 18 |

Table S13 The confusion matrix of Bayes in validation set.

|  | Predicted positive | Predicted negative |
| --- | --- | --- |
| Actrual positive | 12 | 1 |
| Actrual negative | 3 | 8 |
